# Poisoning by Sulphuric Acid

**Published:** 1832-07

**Authors:** Alfred Swaine Taylor

**Affiliations:** Lecturer on Medical Jurisprudence in Guy's Hospital. 35, Great Marlborough street


THE LONDON
### Medical and Physical Journal.

401, VOL. LXVIII.] JULY 1832. [73, *New Series.*

"For many fortunate discoveries in medicine, and for the detection of numerous errors, the world is indebted to the rapid circulation of Monthly Journals; and there never existed any work to which the Faculty, in Europe and America, were under deeper obligations than to the 'Medical and Physical Journal of London,' now forming a long but invaluable series."—RUSH.

### ORIGINAL PAPERS.

### POISONING BY SULPHURIC ACID.

*To the Editors of the London Medical and Physical Journal.*

GENTLEMEN: Perhaps the following details of a case of poisoning by sulphuric acid will prove interesting to those of your readers, whose attention has been directed to the cultivation of the science of Medical Jurisprudence.

M— C—, a servant girl, ætat. nineteen, was brought to an infirmary in my neighbourhood, on Saturday, June 2d, about one P.M., labouring under the following symptoms: great difficulty of deglutition and of speaking; tongue and mouth thickly covered with a white incrustation; oppressed respiration; pulse small, frequent, and somewhat hard; mental faculties clear; no pain complained of, except on pressure in the region of the scrobiculus cordis. The girl obstinately refused to give any account of herself, and those who conveyed her to the infirmary were perfectly ignorant of the cause of her symptoms. A friend of mine, who saw her immediately on her arrival, having some suspicion on his mind, proceeded to the house whence she had been brought, in order to make further inquiries. On examining the bedclothes, they were found to be much soiled and damp to the feel. Those parts which were coloured *blue* had become *red*, and upon applying the tongue, it was found that the wet portions were acid.

Magnesia was now freely exhibited; leeches and fomentations were applied to the abdomen; but the girl continued to sink, and died at five P.M., four hours after her admission. Shortly before her death, she confessed, upon being closely questioned, that she had taken a quantity of common vitriol, but did not assign any reason for her conduct.

*Sectio cadaveris*, forty-two hours after death. Body externally healthy looking. A few dark-brownish spots were to be seen about the mouth and lips, which had proceeded from the action of the acid vomited. The mouth and fauces internally were covered with a thick white incrustation.

The viscera of the thorax were found to be perfectly healthy; œsophagus much destroyed by the acid, but its lining membrane not greatly inflamed.

In the abdomen, the stomach presented externally a highly inflamed appearance, especially towards the upper and posterior part of the cardiac extremity. The redness upon the anterior surface between the two curvatures was disposed in circular bands. Upon laying open its cavity, it was found to contain about three ounces of a dark, viscid, grumous fluid, which was set apart for analysis. The internal surface of the membrane was also highly inflamed, especially about the œsophageal opening and cardiac extremity. The whole interior of the organ was intersected and traversed by prominent black lines, the edges of which were of a deep red. This black matter, which was soft and pulpy, and readily removed by pressure or friction, proceeded from the carbonization of the animal substance. The jejunum and upper part of the ileum were tinged of a slight reddish colour, but there was scarcely any corresponding appearance of inflammation internally. A small portion of the ileum was found to be in a state of intussusception. The large intestines, including the rectum, bore no marks of inflammation, either externally or internally. The other viscera of the abdomen were in their ordinary healthy condition.

I did not see the case during life, but, being requested by my friend to conduct the chemical analysis, I proceeded with him to the house to inspect the bed furniture, &c. I observed that the upper part of the gown worn by the deceased, which was of blue cotton, was tinged of a bright red wherever the fluids ejected had fallen. The bed furniture was also in the same way discoloured, and still slightly damp to the feel. I removed a small portion of the lining of the counterpane, and, having divided this into a number of smaller pieces, digested the whole in boiling distilled water. In this way I obtained a clear solution, which was proved to be acidulous by litmus paper. The fluid being divided into two portions, I added to the one a solution of the supernitrate of barytes, and to the other a few drops of acetate of barytes. In both cases, a heavy precipitate fell, which was inferred to be sulphate of barytes, by its being insoluble in any excess of nitric acid.

The seleniate of barytes is also a salt insoluble in an excess of nitric acid; hence, to remove all liability to fallacy, I dried a portion of the precipitate obtained, and heated it for a few minutes in a platina cup, with a portion of fresh burnt charcoal. By this I obtained either a sulphuret or a seleniuret of of barium; and, to distinguish the one from the other, I moistened the powder with a few drops of muriatic acid, when the presence of sulphuretted hydrogen was indicated by the smell as well as by the change produced in bibulous paper, moistened with a solution of a salt of lead. In this manner was it clearly demonstrated by the evolution of the sulphur with hydrogen, that the acid, combined with the precipitate, and existing in the texture of the vegetable substance, was the sulphuric.

The contents of the stomach were strained and filtered, by which a small quantity of a transparent but somewhat coloured fluid was obtained, which, on being tested with litmus, was found to be faintly acid; but, upon the application of the reagents above mentioned, there was not the least indication of the presence of sulphuric acid.

So far, then, the statement of the deceased was borne out by the result of the chemical analysis; but it yet remains a mystery where she procured the quantity which she must have swallowed; and the vessel* out of which she took it has not yet been found. The following, however, are a few of the facts which I have ascertained: she was seen by her mistress, in apparently good health, on Friday evening at eleven P.M.; but not rising at the usual hour on Saturday morning, her mistress knocked at her door, which, after a time, deceased opened: (this was about seven A.M.) She appeared very ill, and still had on the dress which she wore the preceding evening; but did not state any thing to make her mistress suspect the cause of her illness. A medical man was sent for, who, upon his arrival, recommended her instant removal to an infirmary or hospital; but, for some unaccountable reason, three or four hours elapsed before she was brought in, when she was attended to as already stated.

I will conclude the account by making one or two remarks on the case. The acid swallowed was not in its most concentrated state: this was proved by its not having corroded or charred the white parts of the linen. The quantity taken is unknown; but I think it must have been considerable, from the fact of the whole of the bedclothes being more or less wet. It is probable that the greater part was soon ejected by vomiting; but there were no further attempts at vomiting after she was

* A six-ounce phial, which had contained vitriol, has since been met with.—EDITORS.

discovered. The time at which it was taken is also unknown; but there is reason to believe that it was soon after her mistress left her on the preceding evening, about eleven P.M. Hence this will carry the time for the duration of the case to about eighteen hours, which is about the ordinary period, where the quantity swallowed is considerable.

There are two circumstances in this case worthy of the notice of the medical jurist. The first is connected with the evidence of poisoning from symptoms. It is quite clear, that the medical practitioner who was first called to this case had no suspicion of the cause from which the symptoms originated; or otherwise he would not have ordered her to be conveyed elsewhere, but have applied the remedies himself on the spot. It is true that the girl would confess nothing; but still a proper degree of cross-examination would have elicited sufficient to have excited suspicion, especially when it was known that she had retired to bed in perfect health the night before; when the bedclothes were completely soiled, not to say reddened, by the discharges, and the tongue and fauces covered with a thick white incrustation. Without entering further into these points, I may remark that the result of this case should teach us to be very cautious in forming our diagnosis.

The other circumstance to which I allude is connected with the chemical analysis. Not a trace of the poison was found in the stomach; a result for which I was prepared, since I had already provided myself with part of the bed-linen for analysis, before the inspection was made. The fact is, the mineral acids are not, like arsenic, mercury, or other heavy metallic poisons, capable of being locked within the folds of the mucous membrane: they are fluid, and hence are readily ejected by vomiting. It is on this account that they are but rarely to be found within the stomach, unless death take place very speedily; and that witness who would trust to the chemical analysis of the contents of this organ alone for the detection of these bodies would, in the majority of cases, be altogether deceived.

I have the honour to be, Gentlemen,

Your very obedient servant,

ALFRED SWAINE TAYLOR,

Lecturer on Medical Jurisprudence in Guy's Hospital.

35, *Great Marlborough street;*

*June 6th,* 1832.

